# Hybrid framework of fatigue: connecting motivational control and computational moderators to gamma oscillations

**DOI:** 10.3389/fnrgo.2024.1375913

**Published:** 2024-05-28

**Authors:** Lorraine Borghetti, Taylor Curley, L. Jack Rhodes, Megan B. Morris, Bella Z. Veksler

**Affiliations:** ^1^Air Force Research Laboratory, Wright-Patterson Air Force Base, Dayton, OH, United States; ^2^ORISE at Air Force Research Laboratory, Wright-Patterson Air Force Base, Dayton, OH, United States; ^3^BAE System at Air Force Research Laboratory, Wright-Patterson Air Force Base, Dayton, OH, United States; ^4^Tier1 Performance Solutions at Air Force Research Laboratory, Wright-Patterson Air Force Base, Dayton, OH, United States

**Keywords:** vigilance, fatigue, motivational control, microlapses, computational modeling, high frequency gamma oscillations

## Abstract

**Introduction:**

There is a need to develop a comprehensive account of time-on-task fatigue effects on performance (i.e., the vigilance decrement) to increase predictive accuracy. We address this need by integrating three independent accounts into a novel hybrid framework. This framework unites (1) a motivational system balancing goal and comfort drives as described by an influential cognitive-energetic theory with (2) accumulating microlapses from a recent computational model of fatigue, and (3) frontal gamma oscillations indexing fluctuations in motivational control. Moreover, the hybrid framework formally links brief lapses (occurring over milliseconds) to the dynamics of the motivational system at a temporal scale not otherwise described in the fatigue literature.

**Methods:**

EEG and behavioral data was collected from a brief vigilance task. High frequency gamma oscillations were assayed, indexing effortful controlled processes with motivation as a latent factor. Binned and single-trial gamma power was evaluated for changes in real- and lagged-time and correlated with behavior. Functional connectivity analyses assessed the directionality of gamma power in frontal-parietal communication across time-on-task. As a high-resolution representation of latent motivation, gamma power was scaled by fatigue moderators in two computational models. Microlapses modulated transitions from an effortful controlled state to a minimal-effort default state. The hybrid models were compared to a computational microlapse-only model for goodness-of-fit with simulated data.

**Results:**

Findings suggested real-time high gamma power exhibited properties consistent with effortful motivational control. However, gamma power failed to correlate with increases in response times over time, indicating electrophysiology and behavior relations are insufficient in capturing the full range of fatigue effects. Directional connectivity affirmed the dominance of frontal gamma activity in controlled processes in the frontal-parietal network. Parameterizing high frontal gamma power, as an index of fluctuating relative motivational control, produced results that are as accurate or superior to a previous microlapse-only computational model.

**Discussion:**

The hybrid framework views fatigue as a function of a energetical motivational system, managing the trade-space between controlled processes and competing wellbeing needs. Two gamma computational models provided compelling and parsimonious support for this framework, which can potentially be applied to fatigue intervention technologies and related effectiveness measures.

## 1 Introduction

Vigilance is the ability to sustain attention and remain alert to avoid performance errors on monotonous tasks over time periods ranging from minutes to hours. Air traffic controllers, fire command center personnel, cyber analysts, and airport baggage screeners, among others, routinely engage in monotonous tasks in which performance declines can lead to undesirable personal consequences (e.g., lost productivity) or deleterious organizational outcomes (e.g., industrial accidents or delayed emergency responses). Other empirical findings (e.g., increased performance variability, slowing response times) putatively share the same underlying processes as real world effects (Parasuraman and Davies, 1977; Doran et al., 2001; Dorrian and Dinges, 2004). Such performance declines, i.e., the vigilance decrement, can be traced to an increasingly fatigued cognitive state (Davies and Parasuraman, 1982; Warm et al., 1996). Following Boksem and Tops (2008), we define mental fatigue (hereafter simply referred to as fatigue) as the subjectively experienced feeling, often characterized by tiredness, aversion, and cognitive decline, during or after prolonged periods of cognitive activity. Moreover, mechanisms explaining effects of extended time-on-task have also been linked to sleep loss (Hockey, 1997; Gunzelmann and Veksler, 2018; Veksler and Gunzelmann, 2018), emphasizing the need for comprehensive theories of fatigue given the sleep-challenged pace of modern life (e.g., Webb and Herzog, 2017).

Though the precise causal factors remain under investigation, fatigue ostensibly emerges from interactions between complex cognitive processes taxing information processing, motivation, and affective state (Hockey, 1997; Boksem and Tops, 2008). This complexity becomes apparent when considering the myriad of observed and theorized fatigue effects including: induced stress and aversion to further task investment (Warm et al., 2008), deterioration in cognitive function, e.g., sustaining attention, planning, strategy adaptation (Boksem and Tops, 2008), reduced alertness and increasing lapses in central cognition (Gunzelmann et al., 2009b; Veksler and Gunzelmann, 2018), and compensatory costs such as increased mental effort and sympathetic dominance characterized by physiological discomfort and affective strain (Hockey, 1997, 2011).

A prominent set of theoretical perspectives, collectively known as resource theories, attribute fatigue effects to the depletion of cognitive resources (Caggiano and Parasuraman, 2004). Accordingly, time-on-task drains resources implementing basic information processing functions (e.g., attention, working memory, etc.) which cannot be replenished in the remaining time available (Warm et al., 2008). However, critics argue the mechanisms instantiating a finite pool of resources remain vague and underspecified (Thomson et al., 2015; Inzlicht et al., 2018). Alternatively, cognitive-energetic frameworks view fatigue effects as a function of central control, i.e., the active regulation of basic information processing in the service of goal accomplishment (Hockey, 1997, 2011; Langner and Eickhoff, 2013; Unsworth et al., 2020; Kok, 2022; Schumann et al., 2022). Of particular relevance to the present work, an influential energetical-control theory, the compensatory control model (CCM), implements control as a function of a dynamic motivational system balancing goal-orientation with a competing state of wellbeing (Hockey, 1997, 2011).

While the CCM examines motivation in-depth, the cumulative effects of fatigue on control remain underspecified. By comparison, a recent computational model of fatigue (CMF)—developed in the cognitive architecture Adaptive Control of Thought-Rational (ACT-R; Anderson, 2007)—implements cumulative effects through computational moderators (Veksler and Gunzelmann, 2018). Specifically, increasing time-on-task results in disruptions to central control and related break-downs in goal-oriented processing. With fatigue, gaps known as *microlapses* increasingly occur during information processing, slowing cognition and impairing performance. However, the CMF ascribes motivation a composite role in the vigilance decrement, without regard to competing wellbeing needs.

Though the CCM and CMF, respectively, provide well-specified and empirically validated accounts of fatigue, their differences exemplify dissociations between theoretical frameworks and computational explanations. Computational models of fatigue rarely parameterize motivation explicitly, and cognitive-energetical theories lack specificity with respect to accumulating fatigue. Moreover, neurophysiological evidence supports the energetical perspective but is seldom interpreted in light of specific theoretical models or computational mechanisms, generating additional dissociations between explanations of neural behavior and psychological processes. To resolve these dissociations, we developed a hybrid framework of fatigue which connects the CCM's theoretical perspective with the CMF's computational moderators and changes in neural activity over time. To affirm the framework, we incorporate high frequency gamma oscillations (~70–100 Hz) as the neural representation of a latent (unobserved) factor, i.e., motivational control, in an adapted computational model of fatigue. Ultimately, converging such diverse accounts of fatigue can deepen our knowledge of time-on-tasks effects—beyond treating each perspective as competitive or *sui generis* (Gunzelmann, 2019)—to establish a comprehensive account of the vigilance decrement.

## 2 Components of the hybrid framework of fatigue

### 2.1 Motivation and the compensatory control model

A central claim of the CCM (Hockey, 1997, 2011) is that the motivational system balances competing human needs to regulate performance when experiencing stress or under high workload. Here, stress arises from a mismatch between task-required and extant cognitive states. While the original theory (Hockey, 1997) does not specify exact relations between stress and fatigue, other theories view the latter as an antecedent of former (Hancock, 1989; Warm et al., 2008). Indeed, the CCM's motivational structure maps coherently to fluctuations in performance observed during continuous effort. More recently, Hockey (2011) acknowledged the theory can be understood as an account of task management from the perspective of fatigue. Consequently, the hybrid framework draws heavily upon CCM's core motivational premises and applicability to the cognitive state when strained by time-on-task.

In the context of task completion under stress, the cognitive state strategically prioritizes goal-oriented performance through the energization of effort but at the expense of a state defined by biological comfort and related positive affect, resulting in trade-offs between on- and off-task states. Consistent with other models of control (Jongman, 1998; Carver and Scheier, 2002; Lord et al., 2010), the CCM attributes purposeful behavior and maintenance of active goals to reliance upon a regulatory system referencing internal standards or set points (Hockey, 1997). Such self-regulation manifests as a latent dual-purpose motivational system. Goal-oriented motivation modulates the on-task state through central control, described by Hockey as *motivational control*, while the motivation to remain unencumbered manages the off-task state.

When activated, goal-oriented motivation orients and energizes control given prospective rewards and costs (Hockey, 1997, 2011). While rewards can be extrinsic (e.g., monetary gain), the CCM refers to intrinsic rewards derived from satisfaction in achieving a goal. From this perspective, goal-oriented motivation aligns with innate needs for competence, autonomy, and interpersonal relatedness (Deci and Ryan, 2013). The energizing process intensifies one or more basic cognitive functions, realized as increased *effort*, a key predictor of performance outcomes (Boksem and Tops, 2008; Kok, 2022). We interpret effort mobilization as a function of motivation as implied by the CCM's compensatory process and consistent with Inzlicht et al. (2018), a phenomenological (experienced) rather than an expressed state (Kurzban, 2016; Schumann et al., 2022). However, effort is costly and accompanied by biological activation and subjective strain. Consequently, feelings of stress and aversion evoked in the on-task state conflict with the motivation to be in a leisure and unstressed state. With prolonged time-on-task, the motivational system's control vs. biological and affective functions compete for dominance. Effort must be continually activated, or energized, in order to overcome disruptions from powerful leisure motivations as fatigue sets in.

The CCM conceptualizes motivational control as a compensatory strategy for maintaining task performance in the face of adverse conditions, to include fatigue (Hockey, 1997, 2011). Tensions between the two motivational drives emerge within a control system with lower and upper loops. The lower loop represents the default implementation of well-learned skills aligned with task-relevant performance goals. Effort occurs without appreciable costs and consequently the state remains free from distress. As performance falls below internal reference points for task goals, a problem detecting “effort monitor” determines whether or not to shift control to the higher loop requiring more effort. At this point, the motivational system can either (1) compensate and energize effort, allowing task goals to be protected but at cost of increasing strain and discomfort, or (2) reduce goal aspiration, maintaining wellbeing but accepting a reduction in performance. Both strategies reduce discrepancy between goals and the current state but differentially advantage performance vs. biological comfort. Accordingly, the cognitive system subjectively shifts between the on-task state driven by goal-oriented motivation and the off-task state accommodating wellbeing needs.

The original CCM (Hockey, 1997) does not specify how mechanisms implementing motivational system tensions interact with time-on-task; however, the later version (Hockey, 2011) suggests disruptions to continuous task performance can be viewed as breaks, e.g., blocks, gaps, or lapses. Importantly, breaks from the aversive on-task state refresh effortful control to mitigate performance impairment, even though a build up could occur over time. However, while effects of explicit rests and task changes enjoy considerable attention in the text, the effects of fatigue without these interventions is underexplored.

Related research provides insights into the effects of breaks on cognition and performance over time. A recent review of rest-break literature identifies micro-breaks (<3 min) as brief pauses during continuous cognitive activity allowing for recovery and resulting in performance gains (Schumann et al., 2022). However, absent is an analysis of the extent contexts and processes differ between longer micro-break durations (e.g., 1–3 min) and those consistent with shorter micro-lapses (milliseconds) assumed to accumulate with time-on-task (Gunzelmann et al., 2009a; Veksler and Gunzelmann, 2018). Relatedly, seminal fatigue researcher Bills (1931) theorized about “mental blocks” during extended periods of cognitive work, operationalized as slow reactions more than twice as long as the average across a series. While mental blocks were construed as enforced (i.e., controlled) rests intended to alleviate fatigue and maintain performance over time—consistent with the CCM—the operationalization falls short of accounting for observed performance instability and overall declines in response time in laboratory studies (Parasuraman and Davies, 1977; Dinges and Powell, 1985; Doran et al., 2001). This is conceptually important as microlapse implementation in responses at much shorter time-scales exemplifies the predictive quality of the CMF and the family of models preceding it.

### 2.2 The computational model of fatigue

The CMF implements the accumulating effect of fatigue through well-specified dynamic moderators within the ACT-R architecture (Veksler and Gunzelmann, 2018; see computational model section for technical and mathematical details). These moderators apply time-on-task penalties to the model's current state approximating central control. Unlike its prominence in the CCM, motivation plays a supporting role in the CMF and is parameterized as a composite factor. Motivation, along with other latent factors, such as sleep loss and arousal, are absorbed into an initial expected *utility* value, i.e., the perceived value of a particular action. After task onset, the utility value modulates on/off-task states and degrades as a function of time-on-task and resultant microlapses, i.e., breakdowns in the capacity for focused, goal-directed control. A threshold delineating on-task vs. off-task states simultaneously declines over time as a compensatory strategy to minimize microlapse occurrence and maintain performance.

Historically, vigilance and fatigue have been similarly modeled latent factors as moderators but with less precision. The latent moderator is conceptualized as a state regulator reflecting an individual's goals and affect. These, in turn, change the utility of an action. Importantly, while the latent moderators ostensibly index both arousal and motivation, these terms are neither explicitly defined nor directly connected to psychological theory (Sternberg, 1969; Jongman, 1998).

More recently, earlier CMF versions incorporated models of physiological and psychological fatigue due to sleep loss to modulate latent moderators. An initial line of research by Gunzelmann et al. (2009a; 2009b; 2011; 2015) included the moderator “altertness” directly connected to biomathematical models of circadian dynamics. Here, alertness—the overall readiness of the cognitive system—is estimated from two-process theories of circadian dynamics positing that the human arousal system consists of *circadian* and *sleep homeostatic* components (Jewett and Kronauer, 1999; Hursh et al., 2004; Van Dongen, 2004). The circadian component oscillates across the day, typically with an asymptotic maximum during daylight hours and a minimum during the night. Conversely, the sleep homeostatic component acts as a reservoir that is drained during long periods of wakefulness and is replenished with sleep. These two components interact to generate estimates of alertness that generally decrease during the night and with sleep loss, and increase in daylight hours and with adequate rest.

Such sleep/wake dynamics motivated the conceptualization of fatigue-related microlapses. For example, Doran et al. (2001) theorized that sleep loss led to an unstable cognitive state fluctuating between wakefulness and sleep. Increased sleep loss resulted in lapses in the alert on-task state, where lapses represent a sleepy off-task state. Gunzelmann et al. (2009b) extended this idea to fatigue but recast lapses as briefer microlapses, occurring over tens of milliseconds rather than seconds, minutes, or hours. Microlapses accumulate over time, imposing a penalty on control, resulting in longer off-task duration and diminished performance that cannot be fully overcome by the compensatory threshold mechanism. In data from a simple-reaction task modeled by Veksler and Gunzelmann (2018), microlapse accumulation increased the percentage of slow reactions (defined as longer than 500 ms) from 1% early in the task to over 5% by the end. Here, CMF predictions provided excellent fits to the data. To test generalizability, the model's fatigue mechanisms were further evaluated for a traditional vigilance task as well as for 24 hours of sleep loss with similarly good fits (Gunzelmann and Veksler, 2018; Veksler and Gunzelmann, 2018).

### 2.3 Connecting concepts and mechanisms

Connecting diverse explanations requires new interpretations and instantiations especially for models implemented at different levels of abstraction (CCM, theoretical; CMF, computational). Before doing so, it is important to identify existing convergences between the models: both view fatigue as a function of central control and as a self-regulatory factor rather than a resource, and construe compensatory mechanisms as biologically inspired. These similarities are straightforward and anchor the hybrid framework.

By contrast, interpretations of motivation and fatigue interactions diverge considerably. The hybrid framework reconciles these differences in two ways. First, CMF's alertness fluctuations are interpreted to approximate energetical effort defined by the CCM. This conceptualization aligns with the view that effort can be mobilized by goal-oriented motivation (Inzlicht et al., 2018) while retaining the dynamics of alertness assumed by computational models leveraging biomathematical accounts of fatigue and sleep loss (Gunzelmann et al., 2009a, 2011; Walsh et al., 2014; Veksler and Gunzelmann, 2018). Second, goal-oriented motivation acts as the antecedent of control, providing a latent causal factor for on-task behavior absent in the CMF. When combined in the hybrid framework, we expect motivational control to exhibit properties consistent with cost (aversiveness) vs. benefit (intrinsic achievement) trade-offs of mobilizing effort (Kurzban, 2016; Inzlicht et al., 2018). From the phenomenological perspective, such trade-offs are state dependent, orienting toward homeostatic adaptive behavior, e.g., goal achievement, until after some point in time the cost of persistence (controlled effort) orients the state toward things personally rewarding, e.g., a state of wellbeing or leisure (Kurzban, 2016). Consequently, across time-on-task, we expect an indicator of motivational control to exhibit a relative reduction in influence over the state in comparison to off-task states. Specifically, the utility of effortful control scales according to the accumulation of microlapses. Similar relative value indicators represent dynamic trade-offs, for example, in utilized vs. spare capacity (Steinborn et al., 2017; Schumann et al., 2022) and engaged vs. inattentive states (Mikulka et al., 2002).

Relative fatigue-induced shifts in motivational control can be understood by connecting CMF's microlapses (gaps in control) and CCM's unstressed off-task states. Here, microlapses approximate an off-task state of wellbeing consistent with the default state, where task-unrelated brain activity intrudes upon controlled processes (Langner and Eickhoff, 2013). Similar conceptualizations have emerged in other models, with Unsworth et al. (2020) proposing that moment-to-moment lapses in attention result in brief disengagements from the task that accumulate over the course of a task. Mind-wandering, an off-task phenomenon which increases over time, has been linked to the default state (Kool and Botvinick, 2014; Thomson et al., 2015). In the CMF, transitions to a microlapsed state occur when the cumulative adverse effects of time-on-task impose a penalty on control, resulting in failures to exceed the utility value threshold modulating performance. From the hybrid framework perspective, the CMF's fatigue moderators perform a function analogous to the CCM's “effort monitor” which manages transitions between on- and off-task states. This interpretation allows for the accumulating effect of fatigue on control, the decreasing efficacy of compensatory processes, and dynamic tensions within the dual motivational system.

Assumptions about the dynamical function of control are less easily reconciled. In the CCM, transitions between on- and off-task states reflect adaptive and strategic trade-offs between costs and benefits (Hockey, 1997, 2011). By comparison, the CMF treats fluctuations over time as a general property of cognitive readiness, i.e., alertness (Veksler and Gunzelmann, 2018). While a noise component instantiates alertness fluctuations, the fatigue moderators impose a non-monotonic effect on cognition without regard to strategic intent. Given the efficacy of energetical control as a function of goal-oriented—hence intentional—motivation, the hybrid framework incorporates the CCM's perspective. Here, we use neural data associated with motivation (see next section) to directly model motivational system behavior under fatigue. Through a set of computational models, we demonstrate that parameterizing high frequency gamma oscillations plausibly indexes relative motivational control (i.e., effort) with respect to competing off-task states (i.e., microlapses) and priority shifts over time. Our approach—explicitly modeling motivational control with relevant and temporally-precise neural data—offers a formal means to coalesce previously disparate theoretical perspectives, computational mechanisms, and neural evidence into our hybrid framework of fatigue, as summarized in Box 1.

Box 1 **Key concepts of the hybrid framework of fatigue.**

**Fatigue:** a subjectively experienced feeling, often characterized by tiredness, aversion, and cognitive decline, during or after prolonged periods of cognitive activity; refers to the mental state.

**Motivation:** dynamic self-regulatory system balancing goal-oriented and achievement desires with default needs for affective and biophysical wellbeing; drives energetical control; also referred to as a latent (unobserved) factor in cognition.

**Control:** active regulation of basic information processing functions (e.g., attention, working memory, inhibition, discrimination) in the service of goal-oriented task accomplishment; also referred to as cognitive control, executive control, and central cognition.

**Energetical:** the intensification, via control, of one or more basic information processing functions in meeting some goal; instantiated as brief modulations (milliseconds) within brain networks associated with motivation and control.

**Effort:** the realization of energetical intensification of a basic cognitive function in performance outcomes.

**Microlapses:** brief (tens of milliseconds) breakdowns in focused, goal-directed control; accumulate with time-on-task; analogous to rest/leisure and default states.

**Engagement:** effortful investment in the service of task or cognitive goals; a component of motivation.

**Alertness:** the dynamic cognitive readiness to respond to external information.

**Compensatory control:** attempt to maintain a particular task state under stress; balances goal-directed states with comfortable default states.

**Relative motivational control:** index of effort energized by goal-oriented motivation scaled by microlapses accumulating across increasing time-on-task; implemented as the utility parameter in the gamma computational models.

**High gamma oscillations:** transient high frequency neural oscillations (~70–80 Hz) instantiating intrinsic top-down processes consistent with goal and motivational states.

## 3 Neurological basis of the hybrid framework

Electroencephalography (EEG), assaying oscillatory activity at the millisecond level, enjoys a temporal resolution well-suited to realizing dynamic motivational control. For example, recent work found non-monotonic activity in event-related potentials (ERPs) aggregated across experimental blocks, reflecting pacing and end-spurt effects (Morris et al., 2020) interpretable as a coarse representation of energetical effort over time. Unsurprisingly, non-monotonicity emerged in the frontal N1 ERP associated with controlled attentional orientation, while the parietal P3 ERP, indexing context processing, also exhibited a similar pattern. Other research also demonstrated that non-monotonic ERP activity across diverse vigilance tasks were dominated by frontal ERP components, though energetical activity was also observed in parietal P3 components (Morris et al., 2023). These findings provide converging evidence for the energetical control of basic functions but are less conclusive with respect to cortical orientation and the role of motivation.

In particular, gamma oscillations extracted from EEG data consistently indicate a frontal cortical orientation for energetical control and the involvement of latent motivation. For example, frontal gamma power corresponded to the recruitment of control to maintain goal-oriented cognition (Martel et al., 2019), predicted high engagement in achievement-oriented tasks (Halderman et al., 2021), indexed intentional actions (Karch et al., 2016), and identified maximally alert states (Oken et al., 2006). These findings align with the broader view of frontal gamma as an instantiation of intrinsic high-level (top-down) neural oscillation modulating selective attention and temporal expectancy consistent with motivational states (Engel et al., 2001). Additionally, gamma oscillations have been associated with denser affective networks (Bosman et al., 2014) where motivation is viewed as an affective state (Kool and Botvinick, 2013; Botvinick et al., 2015; Inzlicht et al., 2015).

High-frequency gamma oscillations (~70–80 Hz), in particular, exhibit properties consistent with the motivational system incorporated into our hybrid framework of fatigue. Notably, Assem et al. (2023) recently demonstrated that higher (vs. lower) gamma oscillations increased in the fronto-parietal network (FPN) and decreased in the default mode network during engagement in a cognitively demanding task. In light of our framework, this can be interpreted as the tension between motivation for control and wellbeing needs when the former is intensified. Analogous high gamma effects were observed in other recent studies, with frontal-oriented high gamma power exhibiting non-monotonicity across five consecutive time periods during a brief vigilance task (Borghetti et al., 2021). Drawing from the CMF (Veksler and Gunzelmann, 2018), this dynamic activity was interpreted as a representation of centrally-controlled sustained attention. By contrast, slower frontal beta oscillations (15–30 Hz) increased monotonically across the task, a putative compensatory shift boosting central control (Tran et al., 2020). While increasing power in frontal beta, theta (3–8 Hz), and alpha (9–14 Hz) activity is most frequently linked to compensatory processes, fluctuations in frontal gamma power suggest compensation can separately emerge in the energetical form described by the CCM (Hockey, 1997, 2011). A follow-up study found that gamma connectivity flowed from the frontal to the parietal region as part of a FPN that weakened with time-on-task (Borghetti et al., 2022). Such network attenuation corresponds to the accumulation of microlapses in control consistent with the computational moderators incorporated into our framework.

### 3.1 The hybrid framework and high gamma predictions

Our hybrid framework of fatigue unites the explanatory advantage of a renowned theoretical perspective with the rigorous formalism of a computational model harnessing related neurophysiological data. Specifically, the hybrid framework integrates the CCM's concept of trade-offs between motivational control as a driver of energetical effortful on-task states and unstressed off-task states with the CMF's instantiation of microlapses as a function of aversion to centrally-controlled effort. These processes are viewed as dynamic (fluctuating over time due to competing motivations), compensatory (boosts in effort in response to fatigue effects), and vulnerable to the build-up of time-on-task effects (an increasing desire to remain in an off-task state). We connect these conceptualizations by parameterizing high gamma oscillatory activity to index effortful motivational control in our computational models. To compute relative motivational control under fatigue—reflecting the CCM's cost-benefit dynamics—we adapt the utility parameter from the CMF to moderate goal-oriented motivation. Here, utility values scale the gamma parameter by microlapse accumulation to capture the behavior of competing motivational drives over time.

Before introducing our computational model, we evaluate the extent to which gamma power is associated with changes in behavior. Specifically, computational moderators only become necessary when key observed indicators of fatigue, i.e., gamma activity and response time (RT), do not systemically account for the range of the time-on-task effects. In this case, parameterizing latent variables within computational models can offer a more comprehensive and testable account of underlying processes. Alternatively, computational mechanisms are not needed when expected decrements in RTs across time correspond with changes in related neural activity over time. Using data collected from the PVT, a well-validated simple reaction-time task (see Method section for details), we inspect RTs for decrements with a special emphasis on cumulative distributions and the slowest percentiles, given their heightened sensitivity to time-on-task (Robison et al., 2021; Schumann et al., 2022). To evaluate temporal relations between neural activity and behavior, we correlate gamma power with RTs. To rule out potential confounds to time-on-task effects, we also examine lagged activity and context provided by foreperiods. In other work, lagged, or sequential effects (i.e., enhanced gamma power in a previous trial) predicted improved performance in the next trial (Weissman et al., 2006). We also examine interactions between foreperiod and RTs, as the length of time preceding a stimulus can effect underlying cognitive processes (Langner et al., 2010; Steinborn et al., 2017). Finally, we extend an earlier analysis of FPN dynamics (Borghetti et al., 2022) to investigate potential energetical patterns in the network over time as well as examine relations between network strength, performance, and frontal gamma power.

## 4 Method

### 4.1 Procedure and materials

#### 4.1.1 Participants

Thirty-four individuals (*M*_*age*_ = 22.60; *SD*_*age*_ = 4.08) from a midwestern university and surrounding community participated in the study for monetary compensation. The study was approved by the institutional review boards of the Air Force Research Laboratory and University of Dayton. All participants provided written informed consent in accordance with the Declaration of Helsinki.

#### 4.1.2 Psychomotor Vigilance Test

**Figure 1:**
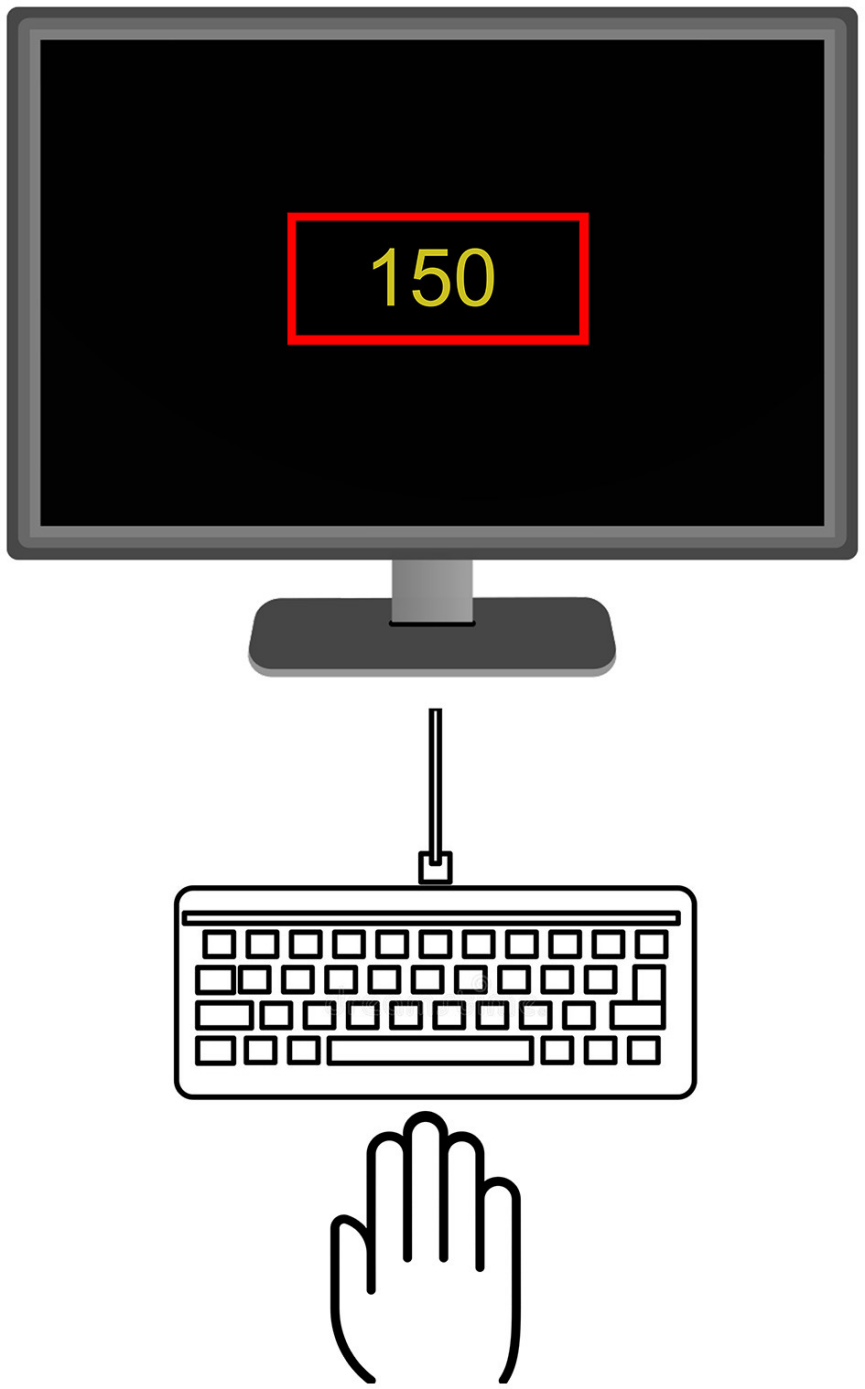
Psychomotor Vigilance Test (PVT). Participants press a pre-designated keyboard button as soon as the numeric counter (center) is detected.

The 10-min Psychomotor Vigilance Test, or PVT (Dinges and Powell, 1985), is a simple reaction-time task measuring changes in attention to a visual stimulus over time. The PVT is the gold standard for measuring sustained attention changes from sleep deprivation/restriction and circadian misalignment (Basner et al., 2018), but also shows time-on-task fatigue effects (Gunzelmann et al., 2010). In the current study, participants monitored a screen with a 5 × 1 cm white, centered box for a visual stimulus, a white numeric millisecond (ms) counter (see Figure 1). At the beginning of each trial, the count started at 1 ms. Participants pressed the “J” key as soon as the count was detected. An interstimulus interval (ISI) (after pressing key) was randomized at discrete second levels between 2 and 10 for each trial. If participants pressed the key before the stimulus, it was recorded as a false start. If participants did not press the key within 30 s after stimulus appearance, it was noted as a time-out [i.e., a sleep attack (Dorrian et al., 2004)]. Because only a response for target detection is required, the PVT avoids confounds for gamma activity potentially present in more complex tasks. For example, the task's low cognitive load constrains gamma to representing controlled attention (Kim et al., 2017). Further, since gamma oscillations exhibit sensitivity to discrimination (Herrmann et al., 2010), using the same stimuli (the counter) in every trial eliminates issues with dissociating sustained attention from target vs. non-target processing.

#### 4.1.3 Data collection and processing

Participants were fitted with a BioSemi ActiveTwo system (BioSemi, Amsterdam, Netherlands, Europe) EEG cap containing 64 Ag-AgCl sintered electrodes following the 10-20 system, with two additional flat, unlinked electrodes applied to the mastoids. Voltage offsets were reduced to <4± 40 mV and noisy channels were re-gelled and re-applied. Scalp activity was re-referenced online to the linked CMS (common mode sense) and DRL (driven right leg) electrodes circuit during recording on a 64 bit Dell PC (AMD Phenom 2.3 GHz processor, 2 GB RAM, Windows 7).

Next, data were cleaned and processed with in-house scripts in MATLAB (version R2019, Mathworks, Massachusetts, USA) and the EEGLAB toolbox 2019.1 (Delorme and Makeig, 2004). Data were downsampled to 512 Hz as needed. A 1.0 Hz high pass filter was applied and the data was re-referenced to the common average. The default EEGLAB independent components analysis (*runica*) was used to identify and remove artifacts due to eye blinks/movement, myogenic noise, and other non-neural signals. Continuous data were epoched into segments ± 1,500 ms with respect to stimulus onset.

### 4.2 Data analysis approach

Gamma power and RTs were analyzed at the aggregate block and single-trial levels. For the block analysis, data were divided into five 2-minute time bins: 0–2 min Bin 1), 2–4 min (Bin 2), 4–6 min (Bin 3), 6–8 min (Bin 4), 8–10 min (Bin 5). Linear mixed-model and correlational analyses were performed using R (R Core Team, 2021). For Tukey contrasts with the holm method, we used the *multicomp* package (Hothorn et al., 2008). Given the potential for sequential effects, we performed a cross-correlation analysis on the single-trial data using the *funtimes* package (Lyubchich et al., 2023). The package's ccf_boot function estimates Pearson correlation coefficients for lagged gamma power (*k-1*) and current trial RTs (*k*) which indicates the extent increases in energy in previous trials predict better performance in subsequent trials. To account for potential autocorrelation, the data is bootstrapped 1,000 times. Cumulative probability distributions were computed using base R's ecdf function.

#### 4.2.1 Response time

For the statistical analysis, RTs were sorted by response type based on historical use of the PVT (Dorrian et al., 2004; Gunzelmann et al., 2009b): alert responses (150–500 ms; 94.3% of responses), false starts (2 instances), and lapsed responses (longer than 500 ms; 5.6% of total responses). In comparison to lapsed responses, alert responses reflect state variability that fluctuates within tens of milliseconds—consistent with microlapses (Gunzelmann et al., 2009b) rather than seconds characteristic of break-rest cycles (Schumann et al., 2022)—to gradually stretch RT distributions to the right. For the time bin analysis, RTs were reduced to a single mean data point for each time bin and aggregated for analysis. To reduce the skew inherent to mean RT distributions, an inverse transformation was applied (*1/RT* × *1,000*; Ratcliff, 1993). RT distributions were examined by rank-ordering each participant's RTs, apportioning them into quintiles from fastest to slowest percentiles (Robison et al., 2021), and then comparing performance across time and quintile.

#### 4.2.2 Gamma power spectral density estimates

Gamma power spectral density (PSD), or power, was estimated in MATLAB. We assayed high gamma power (70–80 Hz) within a window from −300 to 800 ms from the larger epochs at canonical frontal (Fz) and parietal (Pz) sensor locations. The resulting 1100 ms windows were divided into overlapping segments using periodic 367 ms Hamming windows with 25% overlap. Spectral power was next estimated using the pwelch function over the range 0–256 Hz across 2,560 linearly spaced values. Total power was computed by summing single-trial gamma power across subjects within each time bin. To meet the normality assumption, gamma power was log transformed prior to statistical analysis. Consistent with the CMF implementation (Veksler and Gunzelmann, 2018), gamma power data from all trials not rejected for excessive noise in EEG preprocessing were included in our computational models.

#### 4.2.3 Directional connectivity

Here we extend the time Bin 1 vs. Bin 4 Granger Prediction (GP) connectivity findings presented in a recent conference paper (Borghetti et al., 2022) through aggregating all trials in Bin 1 through Bin 5, thus increasing statistical power (at the expense of temporal resolution across time bins), yielding broad though robust differential patterns of frontal ↔ posterior gamma connectivity.

GP, also known as Granger Causality (Granger, 1967), is a neuroscience tool for predicting activity at a given sensor (sensor *X*) from previous activity at sensors *X* and *Y*. The strength of this method is based upon the previous states of *X* and *Y* having greater predictive power for the current state of *X* than does the previous state of *X* alone (e.g., Seth et al., 2015). Any non-zero GP value suggests correlated activity (i.e., directional communication network) between *X* and *Y*. Importantly, GP cannot imply causality in a strict sense between activity at sensors *X* and *Y* (hence the preferred use of “Granger Prediction” over “Granger Causality”), GP is often used to infer causal relations in directional brain networks (e.g., Seth et al., 2015; Winkler et al., 2015).

To investigate frontal ↔ posterior gamma connectivity networks, we used the same single frontal (Fz) and parietal (Pz) sensors as in the power analyses. Frontal → posterior (Fz → Pz) network communication was assessed separately from the posterior → frontal (Pz → Fz) network according to Cohen's (2014) method implemented as described in Borghetti et al. (2022). We calculated GP for Fz → Pz and Pz → Fz for each subject, using EEG data from all trials in Bin 1 through Bin 5. The GP model time window was set to 300 ms with an order parameter of 27 ms, and GP was calculated across 41 log-spaced bins covering 35–100 Hz, and from 250 ms prior to stimulus onset through 1,250 ms after stimulus onset. Specific time windows (early, 0–150 ms after stimulus onset associated with perceptual gating; late, 200–400 ms indexing central cognition) and frequencies (70–80 Hz) of interest were derived from inspection of single-subject and aggregate data from the full time/frequency range described above. Each subject's data was reduced to a single data point (the mean) for each time/frequency window. Further, to explore any brain-behavior linkage between GP and RTs, we examined correlations between these factors separately for each time window and connectivity direction (Fz → Pz, Pz → Fz).

## 5 Results

### 5.1 Gamma power and response time analysis

**Figure 2:**
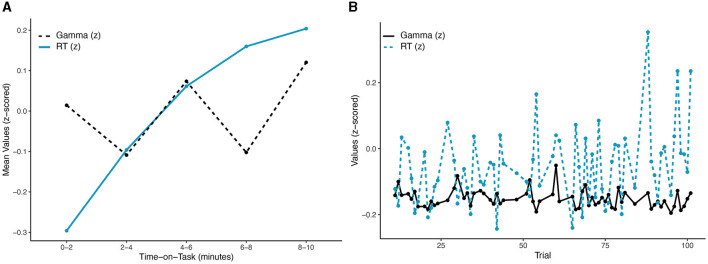
Patterns in gamma power (blue solid line) and RT (black dotted line) over time: **(A)** across the five 2-min time bins (error bars omitted for clarity); **(B)** across single trials. No significant correlations emerged at either timescale.

Behavioral data and neural activity were evaluated for sensitivity to time-on-task as well as interactions with foreperiod, changes in slowest-to-fastest RTs, and correlations in real-time and lagged-time. While anticipating changes across time, systematic temporal associations between the two variables were not expected to emerge. An obvious difference is apparent in Figure 2. The non-monotonic pattern of mean gamma power by bin over time can be visually observed in Figure 2A. These differences were evaluated through a linear mixed model analysis, with a significant effect of bin observed for gamma power, *F*_(4,2525)_ = 4.125, *p* = 0.002, *R*^2^ = 0.77. However, only differences between Bins 2 and 4 vs. Bin 5 survived holm correction for multiple contrasts. The distinct monotonically increasing pattern of RTs across bins in Figure 2A was also significant, *F*_(4,2525)_ = 28.14, *p* < 0.001, *R*^2^ = 0.291. After holm correction, significant contrasts were observed between all bins except Bins 3 vs. 4 and Bins 4 vs. 5, largely consistent with the expected decrement.

A linear-mixed model analysis was performed to evaluate interactions between time-on-task and foreperiod context (i.e., 2–10 s ITIs) on RTs. The ITI-only model was significant, *F*_(9, 2, 520)_ = 28.44, *p* < 0.001, *R*^2^ = 0.33. The ITI + time-on-task model without the interaction was also significant for foreperiod, *F*_(9, 2, 516)_ = 29.97, *p* < 0.001, and time bin, *F*_(4, 2, 516)_ = 34.78, *p* < 0.001, with total *R*^2^ = 0.37. However, the interaction model was not significant, *F*(36, 2,480) = 0.957, *p* = 0.573, *R*^2^ = 0.37. The three models were then evaluated for goodness of fit with the data. The fit for the ITI + time-on-task model was improved over the ITI-only model, χ^2^ (4, *N* = 2,563) = 138.893, *p* < 0.001; however, the interaction model did not improve fit, χ^2^ (36, *N* = 2,563) = 34.818, *p* = 0.547. The results indicate that context provided by varying foreperiod lengths was additive though did not alter the influence of time-on-task on RTs. We do not believe the absence of interaction effects was due to the 10-min duration of the task as the same results were obtained (but not reported in the manuscript) in a study using a 30-min PVT (personal communication, Veksler and Gunzelmann, 2018).

**Figure 3:**
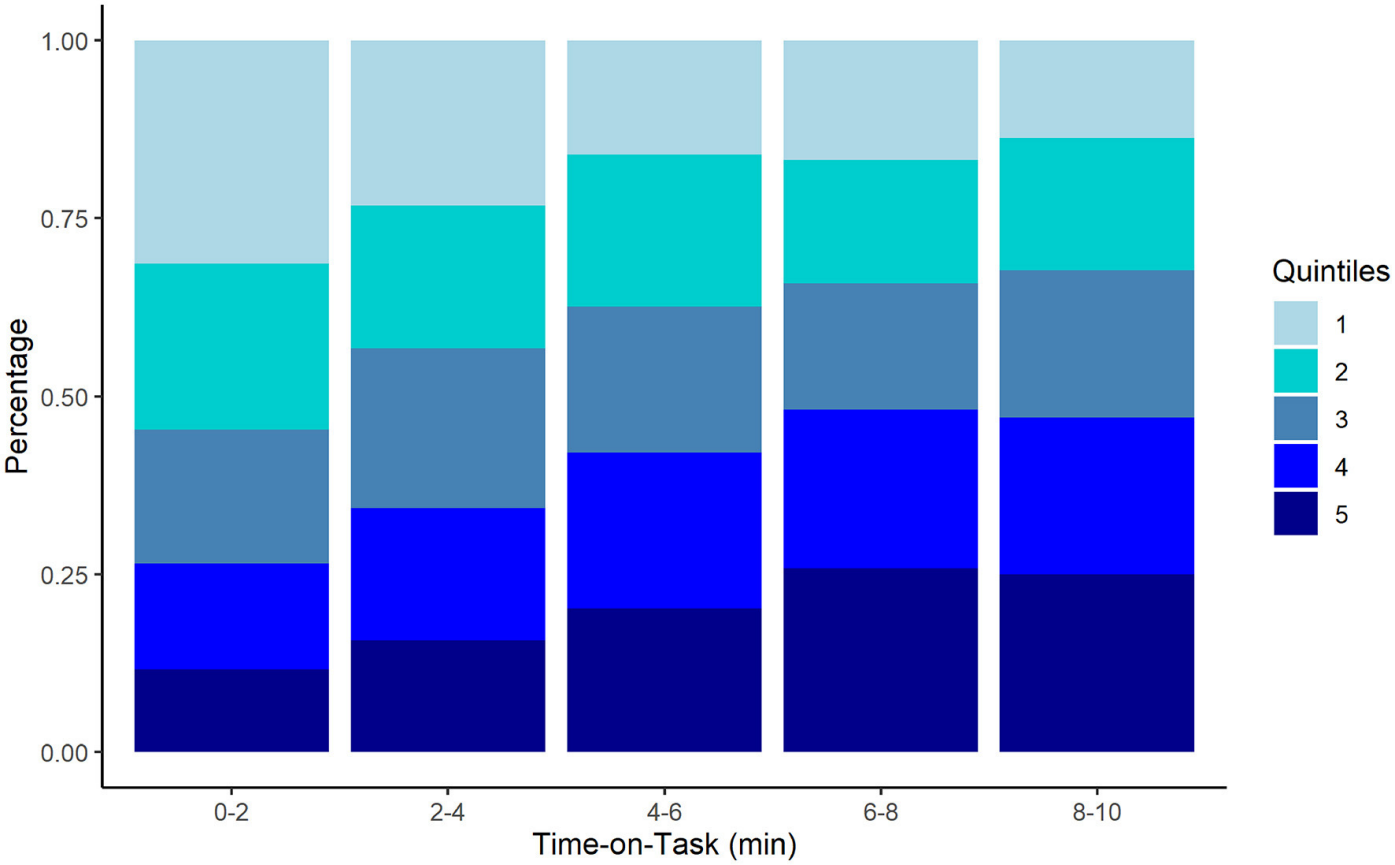
Stacked aggregate counts of fastest (Q1) to slowest (Q5) response time quintiles computed per participant across the five time bins during the 10-min PVT task. Consistent with the vigilance decrement, frequency of the fastest quintile decreases as frequency of slowest quintile increases with time-on-task.

We evaluated change in percentiles over time as fatigue effects have been observed most frequently in the slowest RT percentiles (Robison et al., 2021; Schumann et al., 2022). First, CDF percentiles were divided into quintiles (fastest to slowest) for each participant, then counts for each quintile were computed for each time bin and aggregated. The resulting pattern showed a demonstrative increase in slow responses (Q4, Q5) with time-on-task whereas the number of fastest responses (Q1) declined (see Figure 3; Table 1). Chi-square analysis revealed the change in RT quintile counts over the five time bins was significant, χ^2^ (16, *N* = 2,563) = 91.502, *p* < 0.001. The results provide fine-grained by-participant evidence of a vigilance decrement. Moreover, the decrease in slowest responses from Q4 to Q5 corresponds to endspurt behavior observed in other fatigue and vigilance tasks (Morris et al., 2020, 2022).

**Table 1:** RT Quintile Frequency Counts.

**Time bin**	**Q1**	**Q2**	**Q3**	**Q4**	**Q5**
0–2 min	155	139	100	86	72
2–4 min	117	95	103	100	89
4–6 min	92	103	115	91	115
6–8 min	85	83	93	106	128
8–10 min	64	93	102	159	108

Frequency of quintile counts of fastest (Q1) to slowest (Q5) response time percentiles provide a per-participant account of time-on-task effects. Increases in Q4 and Q5 counts with time are consistent with the vigilance decrement. The modest decrease in counts from Q4 to Q5 corresponds to the endspurt phenomenon.

Correlations between gamma power and RTs were not expected to sufficiently account for motivation and performance. As predicted, correlation at the binned data level was not significant, *r*(3) = −0.286, 95% CI = (−0.932, 0.798), *p* = 0.64. Similarly, a significant correlation coefficient failed to emerge across subjects and single-trials, *r*(2,561) = −0.002, 95% CI = (−0.037, 0.04), *p* = 0.94 (see Figure 2B). Individual correlations were normally distributed and largely insignificant; however, significant correlations were observed in a small number of subjects (4/34, 11.8%).

A lagged Pearson's cross-correlation analysis was performed on the single-trial data for gamma power at time (*k-1*) and RTs at time (*k*) to evaluate sequential effects. Group-level cross-correlations were not significant, *r*(2,561) = 0.017, 95% bootstrap CI = (−0.068, 0.068). Individual cross-correlation estimates were normally distributed and clustered around zero, *r* = (−0.317, 0.374), 95% bootstrap CI: lower(−0.282, −0.204), upper (−0.282, 0.204), though coefficients for four subjects (11.8%) exceeded CI boundaries. Notably, these participants differed from those reported above.

### 5.2 Directional gamma connectivity

We theorized directional connectivity might exhibit a time-on-task pattern consistent with energetical control for frontal → parietal (Fz → Pz) gamma but not for parietal → frontal (Pz → Fz) gamma. However, no significant across-bin (over time) GP changes were observed for early or late window Fz → Pz or Pz → Fz.

**Figure 4:**
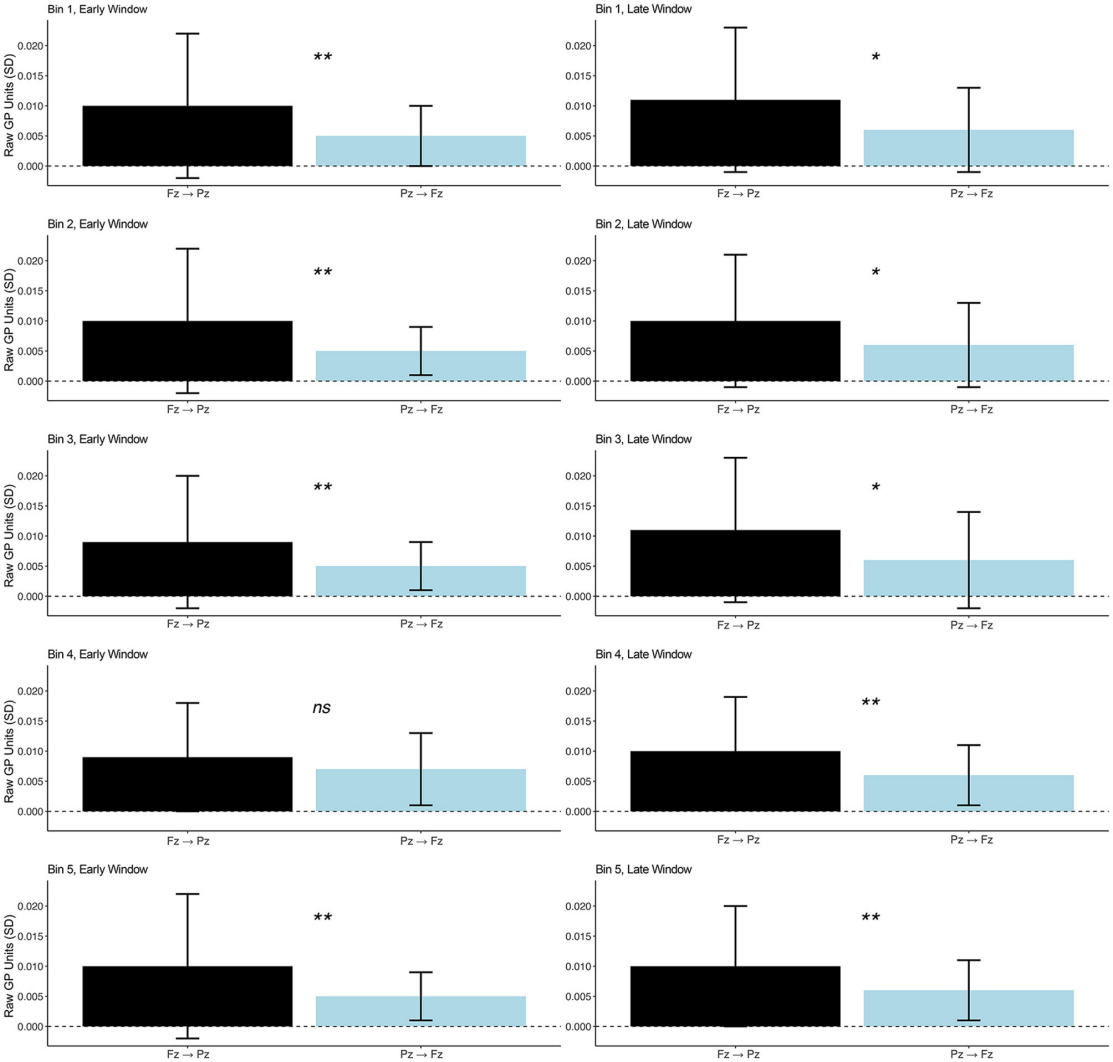
Between-bin high gamma GP for Fz → Pz (black) and Pz → Fz (blue) for an early window (0–150 ms) after stimulus onset associated with perceptual gating and later window (200–400 ms) indexing central cognition; *p ≤ 0.10, **p ≤ 0.05, ns, not significant. Means (*SD*s) are listed in Table 2.

We expected connectivity Fz → Pz gamma to be stronger than Pz → Fz gamma. Indeed, as depicted in Figure 4, we observed greater Fz → Pz than Pz → Fz gamma connectivity in Bins 1, 2, 3, and 5 in the early time window, though there was no difference in Bin 4. Fz → Pz gamma connectivity trended toward being greater than Pz → Fz in Bins 1, 2, and 3 for the late time window and differed significantly in Bins 4 and 5. Table 2 reports *t* and *p* values as well as mean (*SD*) GP by bin and connectivity direction for each time window. The results provide evidence for frontally dominated cognition consistent with energetical control.

**Table 2:** Within-bin GP by direction, bin, and time window.

			**Fz → Pz**	**Pz → Fz**
**Bin**	***t*** **(66)**	***p***	***M*** **(*****SD*****)**	***M*** **(*****SD*****)**
**Early window**				
Bin 1	2.21	0.03	0.010 (0.012)	0.005 (0.005)
Bin 2	2.15	0.04	0.010 (0.012)	0.005 (0.004)
Bin 3	2.13	0.04	0.009 (0.011)	0.005 (0.004)
Bin 4	1.15	0.25	0.009 (0.009)	0.007 (0.006)
Bin 5	2.24	0.03	0.010 (0.012)	0.005 (0.004)
**Late window**				
Bin 1	1.90	0.06	0.011 (0.012)	0.006 (0.007)
Bin 2	1.78	0.08	0.010 (0.011)	0.006 (0.007)
Bin 3	1.74	0.09	0.011 (0.012)	0.006 (0.008)
Bin 4	2.11	0.04	0.010 (0.009)	0.006 (0.005)
Bin 5	2.17	0.04	0.010 (0.010)	0.006 (0.005)

### 5.3 GP correlations with RT and gamma power

As with gamma power, a direct association between GP and performance was not expected. A Pearson's correlation revealed only a marginally significant relation between GP and RT for Fz → Pz connectivity for Bin 1 in the late time window. All other correlations were nonsignificant.

Alternatively, we expected frontal gamma power to exhibit a positive relationship with Fz → Pz GP but not for Pz → Fz GP. As listed in Table 3, correlations between Fz → Pz GP and frontal gamma power were significant in the early time window at Bins 4 and 5. In the late time window, these correlations for Fz → Pz were significant at Bins 2, 3, 4, and 5, though Bin 1 was nonsignificant. All correlations between Pz → Fz GP and frontal gamma power were nonsignificant.

**Table 3:** Correlations between GP and frontal gamma spectral power by direction, time window, and bin.

	**Fz → Pz**	**Pz → Fz**
**Early window**		
Bin 1	*r* = 0.31, *p* = 0.08	*r* = −0.07, *p* = 0.68
Bin 2	*r* = 0.30, *p* = 0.08	*r* = −0.04, *p* = 0.81
Bin 3	*r* = 0.31, *p* = 0.08	*r* = 0.01, *p* = 0.97
Bin 4	*r* = 0.56, *p* < 0.01	*r* = 0.08, *p* = 0.67
Bin 5	*r* = 0.40, *p* = 0.02	*r* = 0.19, *p* = 0.28
**Late window**		
Bin 1	*r* = 0.24, *p* = 0.17	*r* = −0.02, *p* = 0.92
Bin 2	*r* = 0.37, *p* = 0.03	*r* = −0.04, *p* = 0.82
Bin 3	*r* = 0.35, *p* = 0.04	*r* = −0.02, *p* = 0.90
Bin 4	*r* = 0.57, *p* < 0.01	*r* = 0.03, *p* = 0.89
Bin 5	*r* = 0.64, *p* < 0.01	*r* = −0.01, *p* = 0.95

### 5.4 Summary of data analysis results

Our analysis largely supports our hypothesis that high gamma activity, as a representation of motivational control, does not sufficiently associate with behavioral performance to fully account for accumulating latent motivational tensions. A detailed analysis of RT dynamics provides evidence for time-on-task effects beyond foreperiod influences, and validates the increased frequency of participant slowest responses later in the task consistent with the vigilance decrement and end-spurt phenomenon. The absence of bin-level and single-trial correlations indicates the need for additional mechanisms to explain fatigue effects. Sequential effects were also not observed, removing a potential confound for interpreting the moment-to-moment interactions. That a small number of individuals' data was correlated, potentially reflect performance differences indexed by gamma oscillations in previous research (Herrmann et al., 2010; Kim et al., 2017; Borghetti et al., 2021).

The GP analyses provided evidence that neural communication over time maintains a frontal-orientation consistent with a FPN associated with central control. However, in contrast to gamma power, an energetical pattern was not observed in GP between bins. Even so, correlations between oscillatory power and the FPN indicate the suitability of frontal high gamma as a latent driver of information processes necessary to maintain task performance. By contrast, the absence of RT and connectivity further supports the notion that a multitude of cognitive factors contribute to the vigilance decrement (Boksem and Tops, 2008).

Collectively, the findings provide compelling but insufficient evidence for the hybrid framework of fatigue. Frontal high gamma oscillations exhibit an energetical pattern and connectivity corresponding to central control but do not directly map to observed performance declines. Consequently, we feel confident in using gamma power as an index of the motivational system in our computational model.

## 6 Computational model of the hybrid framework

We demonstrate the efficacy of our hybrid framework of fatigue through representing components in a computational model and then testing the model's ability to describe and predict performance. Computational models of human cognition are a common method of mapping empirical observations to theory, where latent psychological constructs are translated into free parameters and fit to empirical data (Pitt and Myung, 2002; Farrell and Lewandowsky, 2018). By managing multiple dynamic interacting components and processes, computational models can handle complex systems, i.e., regulatory control in the human mind, that are challenging to analyze using analytical methods alone.

We introduce two computational models that test the hypothesized relationship between energetical motivational control and accumulating microlapses in a simulated vigilance task. We first present earlier versions of CMF for the PVT and then extend the model to integrate gamma PSD, i.e., high gamma power, as a representation of motivationally-driven control. Motivational trade-offs due to fatigue are captured through utility values computed by scaling gamma PSD by fatigue moderators (time, microlapses). The models are written using ACT-R (Anderson et al., 2004)—a goal-directed, time-dependent architecture with a long history of simulating sustained attention (Gunzelmann et al., 2009a,b; Walsh et al., 2017; Veksler and Gunzelmann, 2018).

### 6.1 ACT-R theory

The ACT-R architecture conceptualizes behavior as arising from interactions between stored knowledge and actions that are used to execute behaviors. In models of simple reaction time tasks, most behavior is related to the selection and execution of specific pieces of procedural knowledge, e.g., goals, which are referred to as “productions” approximating central control (Anderson et al., 2004). The ACT-R architecture conceptualizes behavior as arising from interactions between stored knowledge and actions that are used to execute behaviors. These interactions emerge from a series of if/then rules that govern which actions are appropriate in a given situation. In models of simple reaction time tasks, most behavior is related to the selection and execution of specific pieces of procedural knowledge, e.g., goals, which are referred to as “productions” approximating central control (Anderson et al., 2004).

ACT-R selects the production with the greatest estimated utility value *U*, a value that reflects the strength and appropriateness (via partial matching and similarity) of a given behavior at a given time, to execute (or “fire”) when an action takes place. Conflict resolution refers to the process of selecting from the set of possible productions. The probability of selecting a production is directly related to the estimated utilities of the productions held in procedural memory, noise on these values (σ^2^), and a threshold (*UT*) that filters out productions with low *U* values. Utility noise σ^2^ is selected from a logistic distribution with mean 0 and variance $\frac{\pi }{3}\cdot s^{2}$ (where *s* is a free parameter that controls the magnitude of the variance component) and produces stochasticity in selecting production *i* which is represented mathematically using Luce's (1977) choice axiom as shown in Equation 1:


(1) $$\begin{array}{c} Pr\left[U_{i}\right]=\frac{e^{U_{i}/\sqrt{2}s}}{\sum_{J}e^{U_{j}/\sqrt{2}s}}, \end{array}$$

where the denominator sums the utilities for matching above-threshold productions of length *J*.

### 6.2 Simulations of vigilance and motivation

This goal-directed production system has proven useful for simulations of vigilant performance for a number of reasons. First, it comports with the dynamics of well-known vigilance tasks, such as the PVT and the Mackworth Clock Task (Mackworth, 1948), where participants transition between discrete states in accordance with the task, e.g., “waiting” before a stimulus appears and “responding” after a stimulus has been presented. These transitions are imperfect and are subject to change, as participants show variability in their decisions to withhold or produce a response, particularly as a function of changes in internal (e.g., fatigue, motivation) or external (e.g., visual noise) influences across time. In this way, sustained attention tasks, and cognitive models of such tasks, can be considered time-inhomogeneous semi-Markov processes (c.f., Weaver, 2008; Fisher et al., 2022). This characteristic relates to the second reason, that changes in state transition probabilities (via production utilities) can be easily modeled using a small number of parameters. This can be accomplished either using methods built into the ACT-R architecture, such as production reinforcement (e.g., Lovett and Anderson, 1996), or for the CMF, incorporating a fatigue module (i.e., the fatigue moderators) that directly influences the parameters of the model.

In earlier computational models of fatigue, estimates of alertness were integrated into ACT-R in a way similar to other PVT models (Jongman, 1998; Belavkin, 2001); namely, estimated alertness modulated the expected utility values of relevant productions in the model, where lower alertness estimates resulted in lower utility values and, in return, lower probabilities of a given production occurring (Gunzelmann et al., 2009a). To help characterize performance, more recent models introduced: (1) microlapses, e.g., effects of brief lapses in control, and (2) compensatory efforts to help offset lowered production utility values. Microlapses occur during moments when no model productions exceed the utility threshold (e.g., during states of aversiveness) and result in a short penalty to processing time (~50 ms). Microlapses also result in decreases in production utility values, leading to even more microlapses. Compensatory efforts, on the other hand, are not tied to utility values, and instead affect utility thresholds by lowering them with increased fatigue. This reduces the chance that no productions are selected to fire (resulting in the model “getting stuck”) but increases the probability of the model firing inappropriate productions, such as responding before the stimulus is presented (false alarm) or withholding a response in the presence of the stimulus (lapse). Earlier models connected decreases in performance across time-on-task to time awake primarily using biomathematical models of arousal based on circadian dynamics, such as SAFTE (Hursh et al., 2004; Gunzelmann et al., 2009a,b, 2011; Walsh et al., 2014); however, these effects can be generalized using time-on-task penalties (Veksler and Gunzelmann, 2018).

**Figure 5:**
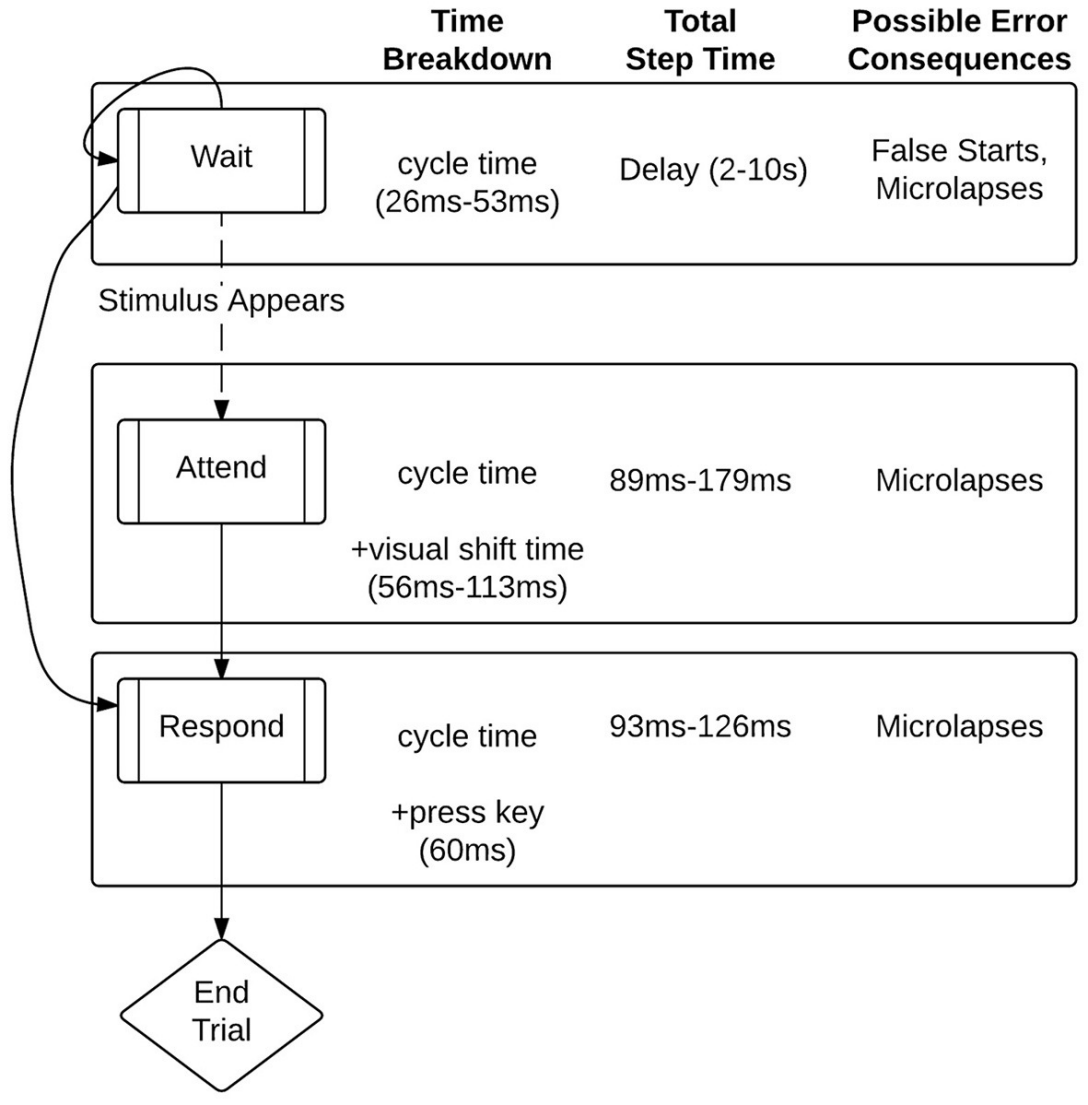
Process model of the Psychomotor Vigilance Test (PVT) in ACT-R. From Veksler and Gunzelmann (2018).

The most recent version of the CMF models effects of fatigue on performance in short (10–30 min) PVT tasks (Veksler and Gunzelmann, 2018). In the model, the state transitions between “waiting,” “attending,” or “responding” (see Figure 5).

Here, alertness is not estimated from biomathematical models of fatigue; instead, it is assumed that alertness decreases as a function of fatigue moderators—time-on-task and microlapses—which approximate the cost of performance at shorter time-scales (i.e., minutes vs. hours and days). Specifically, the authors estimate (Equation 2) the utility of a production *U* by imposing a penalty on the initial production utility value (υ) as a function of both the number of microlapses (*N*_*ml*_) and the time spent on the experiment (*t*):


(2) $$\begin{array}{c} U\left(t\right)=\upsilon \;\left[\lambda^{N_{ml}}{\left(1+t\right)}^{\rho }\right], \end{array}$$

where υ is the initial utility value parameter, *t* is time spent on the task (scaled to minutes), λ scales the effect of micro-lapses on utility values, and ρ scales the effect of time-on-task. As fatigue increases and production values decrease, the probability of sampling an inappropriate production increases, leading to increases in false alarms.

In contrast, the production utility selection threshold is only affected by time-on-task as shown in Equation 3:


(3) $$\begin{array}{c} UT\left(t\right)=\tau \;{\left(1+t\right)}^{\kappa }, \end{array}$$

where τ is the initial utility threshold parameter and κ scales the effect of time-on-task on the threshold. Lower thresholds under conditions of fatigue allow the model to select productions whose υ values have decreased, which can also lead to selecting inappropriate productions, e.g., false alarms.

### 6.3 Current model development

The current effort expands Veksler and Gunzelmann's (2018) CMF in the PVT to instantiate concepts of our hybrid framework of fatigue. As such, the model implements interactions between fatigue, relative motivational control, and the observed patterns in the neural and behavioral data from the current study (c.f. “Results”). Specifically, we modeled the combined influences of fatigue (e.g., slowed responses due to time-on-task) and motivation (e.g., energetical control and effort) on PVT performance across all participants and within individuals. In contrast to previous models, but consistent with our proposed hybrid framework, we define alertness as the composite representation of the subjective intensification of a cognitive function (such as controlled attention) in the service of a goal (Inzlicht et al., 2018). That is, alertness approximates effort consistent with the CCM (Hockey, 1997, 2011). Our model construes motivation as a system that either intensifies (i.e., increases) or constrains (i.e., decreases) performance behaviors. This implementation corresponds to the CCM such that intrinsic goals influence the control of task performance, similar to a hyperparameter in a standard cognitive model, and wellbeing needs extract a cost on performance. We assume that task goals (on-task states) and wellbeing (off-task states) vary across individuals—namely that subjects engage in a subjective cost-benefit calculus—resulting in differences in energy expenditures. Utility values index evolving cost-benefit tensions across time-on-task to account for the waning trade-space advantaging achievement goals due to fatigue.

#### 6.3.1 Parameterized motivation

We instantiate motivation as the parameter zeta (ζ) that is typically bounded between zero and one. In the proposed model, however, ζ can exceed its upper bound, meaning that parameterized effort cannot go below zero (meaning “absolute” fatigue), but can surpass unity (meaning “extra” effort). Thus, ζ can capture decrements due to fatigue (off-task states) as well as compensatory efforts that offset fatigue (on-task states).

**Figure 6:**
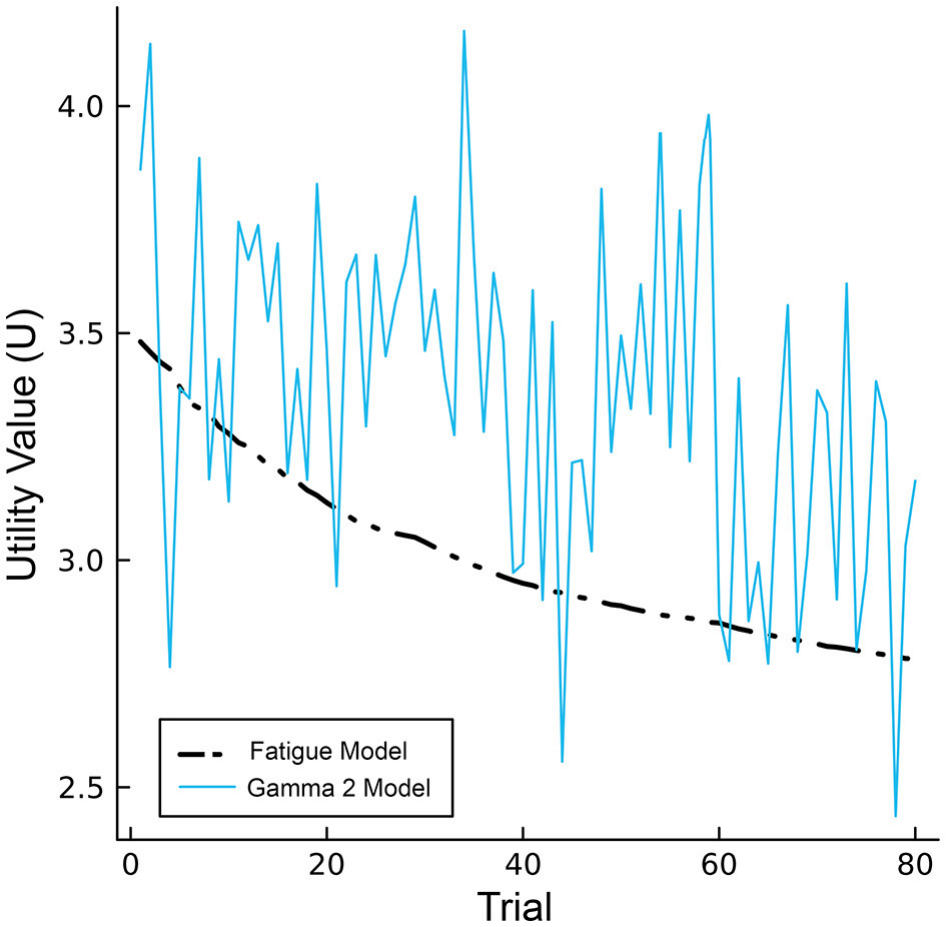
Single-trial utility values for the CMF (black) and Gamma model 2 (blue), excluding noise.

One way to normalize fatigue moderator values is by adjusting the values to the smallest value and the range of the values. This normalization method has been used to scale biomathematical estimates of arousal in previous investigations of the PVT (Gunzelmann et al., 2009b), where estimates start with high values and monotonically decrease as a function of time. An interesting aspect of this method that is reflected in the fatigue moderators proposed by Gunzelmann et al. (2009b); Veksler and Gunzelmann (2018) is that the normalized values start at one (the highest possible value) and decrease with time-on-task, implying that performance cannot meet or exceed that from *t* = 1. Normalizing from the starting value, however, allows the transformed values to reflect the energetical variation from “baseline” (i.e., beginning of the task), including instances where performance exceeds initial efforts (unity). Therefore, we opted to normalize gamma values to a baseline reference in order to simulate pacing and end-spurt effects using gamma PSD estimated across 2-min bins (Model 1) and motivational control within individual trials (Model 2). The resulting values are then multiplied against production utility values to simulate changes in performance. Parameterizing motivation in this way highlights a distinction between the CMF using fatigue moderators and models using interactions between gamma activity and fatigue moderators. Without noise, utility values for CMF exhibit a monotonic decline over time whereas utility values for the gamma models exhibit dynamic and declining properties across the task consistent with hybrid model predictions (see Figure 6).

#### 6.3.2 Model 1: binned gamma

Our first model uses the PSD estimates binned across 2-min time intervals observed in the current study to examine broad trends in cognitive-energetical behavior (Figure 2A). Given a set of observed PSD estimates Γ_*i*_ = {γ_*i*,1_, …, γ_*i,t*_}, for participant *i* at time bin *t*, as well as the range of these values, γ_*r*_*i*__ = range{γ_*i*,1_, …, γ_*i,t*_}, we can calculate effort as shown in Equation 4:


(4) $$\begin{array}{c} \zeta_{i,t}=1+\left(\frac{\gamma_{i,t}-\gamma_{i,1}}{\gamma_{r_{i}}}\right). \end{array}$$

Here, ζ_*i*,1_ = 1 and all subsequent values are interpreted as diminished effort due to time-on-task (ζ_*i,t*_ < ζ_*i*,1_) or as intensified (i.e., compensatory) effort compared to baseline (ζ_*i,t*_ > ζ_*i*,1_), allowing the model to account for pacing and end-spurt effects observed in the data.

#### 6.3.3 Model 2: single trial gamma

We also developed a model of the PVT that examines fluctuations in performance with higher temporal precision using gamma PSD values estimated for each trial. Because of the high variability in these PSD estimates, we used a simple modification of the common decibel conversion method (c.f., Cohen, 2014). As shown in Equation 5, ζ values are calculated as a ratio of gamma power for a given trial (*t*) against a baseline comparison calculated as the average spectral power for trials 1 through *k*, where *k* is typically set to 10. This ratio is then scaled into units appropriate for the simulation by adding 1 to the logarithm of the ratio:


(5) $$\begin{array}{c} \zeta_{i,t}=1+log_{b_{i}}\left(\frac{\gamma_{i,t}}{\mu_{\gamma_{i,1:k}}}\right), \end{array}$$

where *b* is a parameter that allows the base of the logarithm to vary for each participant. The base can be any multiple of 10 that is >10 (the default value).

#### 6.3.4 Applying fatigue moderators

To moderate performance, we integrate parameterized motivation in a linear function with the initial production utility parameter υ (similar to Equation 2) with brief lapses in central control. Therefore, as shown in Equation 6, the modulated utility value for an individual at a given time, *U*_*i,t*_, can be calculated as a function of υ_*i*_, ζ_*i,t*_, and a penalty (λ_*i*_) to the number of simulated microlapses (*N*_*ml*_):


(6) $$\begin{array}{c} U_{i,t}=\upsilon_{i}\cdot \left[\lambda_{i}^{N_{ml}}\cdot \zeta_{i,t}\right]. \end{array}$$

The probability of selecting a given production (wait, attend, respond) then, is positively related to parameterized motivation (ζ), but negatively related to the number of brief lapses in control during the vigil.

### 6.4 Results

We used the neural and response time data observed in this study to examine the ability for the *gamma 1* and *gamma 2* models to simulate performance during the 10-min PVT. Performance in these models was compared against the most recent CMF of PVT performance that includes the additional ρ and κ parameters listed in Equations (2) and (3), respectively (c.f., Veksler and Gunzelmann, 2018).

To reduce the complexity of the models and potential for poor convergence, we fixed the initial utility threshold τ to 2.0 for all participants in all models. Therefore, all models include initial utility value (υ), microlapse penalty (λ), and conflict resolution time (ϕ) as free parameters, with two additional time-on-task penalty parameters for utility values (ρ) and thresholds (κ) in the CMF model (Table 4).

**Table 4:** Descriptions of parameters in the ACT-R models of the PVT.

**Param**.	**Description**	**Bounds**	**Value**	**Gamma models?**
υ	Initial production utility value	(0.0, Inf)	Free	Yes
τ	Initial production utility threshold	*N/A*	2.0	Yes
λ	Microlapse penalty	(0.0, 1.0)	Free	Yes
ϕ	Conflict resolution time	(0.01, 0.1)	Free	Yes
ρ	Production utility time-on-task penalty	(−1.0, 0.0)	Free	No
κ	Utility threshold time-on-task penalty	(−1.0, 0.0]	Free	No

The “Value” column indicates if a value is freely-estimated, and if not, what the value is fixed to. The “Gamma models?” column indicates if the parameter is included in the models that use gamma power as a performance moderator.

The model-fitting procedures consisted of maximizing the likelihood of trial-level latency for each subject using approximate Bayesian computation (ABC) with differential evolution (Turner and Sederberg, 2012). All model code was developed using the Julia programming language (Bezanson et al., 2017) and open source code libraries, including DifferentialEvolutionMCMC.jl (dfish, 2022). The parameters estimated for each of the three types of simulations are listed in Table 5.

**Table 5:** Summary statistics of the parameters from the best-fitting models fit to data from individual participants.

	**Parameters**					**Fit indices**	
**Model**	υ	λ	ϕ	ρ	κ	***-2LL***	***AIC***
CMF	3.82	0.88	0.057	−0.20	−0.15	1,137.63	1,147.63
	(0.14)	(0.01)	(0.002)	(0.01)	(0.01)	(16.55)	(16.55)
Gamma model 1	6.65	0.94	0.058	–	–	1,176.06	1,182.06
	(0.93)	(0.01)	(0.002)	–	–	(21.29)	(21.29)
Gamma model 2	4.15	0.93	0.058	–	–	961.74	967.74
	(0.40)	(0.01)	(0.002)	–	–	(26.03)	(26.03)

The mean values are listed above the standard errors in parentheses.

#### 6.4.1 CMF model

The parameters recovered using the CMF model are consistent with those reported by Veksler and Gunzelmann (2018). Specifically, initial utility values were greater than the fixed threshold parameter, *M*_υ_ = 3.82, *SE*_υ_ = 0.14, while the conflict resolution time was around 50 ms, *M*_ϕ_ = 0.057, *SE*_ϕ_ = 0.002. These results did diverge from the original parameter estimates in two ways, however: First, the two penalties for time-on-task, *M*_ρ_ = −0.20, *SE*_ρ_ = 0.01, *M*_κ_ = −0.15, *SE*_κ_ = 0.01, are much greater in magnitude compared to those estimated by Veksler and Gunzelmann (ρ = −0.05, κ = −0.01). Second, when allowed to freely-vary, the average estimated penalty to microlapses, *M*_λ_ = 0.88, *SE*_λ_ = 0.01, is smaller than the fixed value given by the authors (λ = 0.98). This could be due to differences in lengths of the two PVT tasks (30- vs. 10-min), such that greater time-on-task penalties are needed to account for decrements across shorter periods of time.

#### 6.4.2 Gamma model 1

The model that integrated gamma PSDs estimated in five time bins (i.e., *Model 1*) resulted in a similar pattern of parameters: initial utility values, *M*_υ_ = 6.65, *SE*_υ_ = 0.93, are greater than the fixed initial threshold values, with microlapse penalties close to 0.9, *M*_λ_ = 0.94, *SE*_λ_ = 0.01, and conflict resolution times close to 50 ms, *M*_ϕ_ = 0.058, *SE*_ϕ_ = 0.002. The average fit of the *gamma 1* model to the observed data, *M*_AIC_ = 1176.06, *SE*_AIC_ = 21.29, is no different than that for the CMF model, *M*_AIC_ = 1137.63, *SE*_AIC_ = 16.55, *t*(33) = 1.39, *p* = 0.17, *d* = 0.34, indicating that model's transformations of binned gamma PSD values provide estimates of PVT performance that are as accurate as those given by previously-published models.

#### 6.4.3 Gamma model 2

Finally, when gamma PSD values are transformed on a trial-by-trial basis, the recovered parameters are similar to those captured by the CMF and *Model 1*, but with better fit to the observed data, *M*_AIC_ = 967.74, *SE*_AIC_ = 26.03, compared to the CMF, *t*(33) = 5.67, *p* < 0.05, *d* = 1.37, and *gamma* 1 models, *t*(33) = 6.37, *p* < 0.05, *d* = 1.55. Initial utility values are again greater than the fixed initial threshold values, *M*_υ_ = 4.15, *SE*_υ_ = 0.40, microlapse penalties are close to 0.9, *M*_λ_ = 0.93, *SE*_λ_ = 0.01, and the average conflict resolution time is close to 50 ms *M*_ϕ_ = 0.058, *SE*_ϕ_ = 0.002.

### 6.5 Model discussion

The model presented here demonstrate that fluctuations in performance on the PVT arising from fatigue and motivational control can be represented using high frequency neural activity observed during the task. Critically, declines in utility values over time can be interpreted as the dynamic re-orientation of the state away from goal-motivated control to leisurely off-task states. Here, transformed gamma PSD values reflect the energetical variations from unity (e.g., increases above baseline) in balancing competing motivations. *Model 1* uses gamma PSD values collected in 2-min time bins to moderate transition probabilities between states of waiting, attending, and responding while *Model 2* does the same, but with gamma PSD estimates calculated for each trial. The simulated reaction times suggest that a model integrating binned gamma PSD values (*Model 1*) produces results that are as accurate as the most recent computational model of fatigue (CMF model; Veksler and Gunzelmann, 2018) using fewer assumptions and free parameters. The results also suggest that increasing the temporal resolution of these PSD values, as done in *Model 2*, significantly increases the accuracy of the computational model. These new models allow for individual differences in parameter estimations and for differences in energetical control as well as broader pacing and end-spurt effects in the PVT—patterns not previously captured in a computational model of fatigue. Importantly, *Model 1* and *Model 2* provide effective and parsimonious demonstrations of the integration of cognitive-energetic theory, i.e., the CCF, and computational modeling formalisms, i.e., the CMF, with neural data in computational simulations of the vigilance decrement—satisfying the intent of our hybrid framework of fatigue.

## 7 Full discussion

The current work introduces a novel hybrid framework of fatigue which connects an influential cognitive-energetic theoretical perspective (Hockey, 1997, 2011) with moderators from a recent computational model of fatigue (Veksler and Gunzelmann, 2018) and gamma oscillations representing motivational control. The framework put forth a more cohesive explanation of the vigilance decrement than considering each of the constituent accounts independently. The theoretical perspective, Hockey's (1997; 2011) CCM, emphasizes interactions between competing motivations, energetical control, and effort, but remains somewhat ambiguous about the cumulative effects of fatigue on performance. Alternatively, Veksler and Gunzelmann's (2018) CMF specifies mechanisms, i.e., microlapses, responsible for time-on-task performance declines but does not consider the implications of a motivational system. When united in the hybrid framework, motivational control exhibits properties consistent with cost (aversiveness) vs. benefit (intrinsic achievement) trade-offs mobilizing effort. From the phenomenological perspective, such trade-offs are state dependent, orienting toward adaptive behavior, e.g., goal achievement, until after some point in time, the cost of persistence (controlled effort) orients the state toward to things personally rewarding, e.g., a state of wellbeing or leisure (Kurzban, 2016). As a result, motivational control experiences a relative reduction over time in comparison to off-task states. Moreover, the hybrid framework formally links very brief lapses (occurring over milliseconds) in control to fluctuations in the motivational system at a temporal scale not otherwise described in the fatigue literature.

Frontally-oriented high frequency gamma oscillations instantiate the rapid dynamic interplay between motivation and fatigue. In our computational models, parameterized gamma power modulated interactions between the CCM's dual-purpose motivational system and CMF's moderators implementing accumulating time-on-task effects. Notably, binned and single-trial gamma power along with frontal-parietal connectivity exhibited properties consistent with energetical cognition and motivational control but failed to correlate with behavior to capture the full range of fatigue effects. By contrast, our best fitting computational model, *Model 2* using single-trial neural data, offers compelling and parsimonious support for the hybrid framework's conceptualization of interacting fatigue mechanisms.

To our knowledge, gamma oscillations have not previously been parameterized to index tension between the goal-oriented and default drives of the motivational system under fatigue. While numerous perspectives view gamma power as an index of motivation-related top-down (i.e., controlled) processes (Engel et al., 2001; Karch et al., 2016; Kim et al., 2017; Martel et al., 2019; Halderman et al., 2021), dynamic transitions between peaks (as energized motivational control) and troughs (as gaps in control) remain largely unaddressed. In our hybrid framework, troughs reflect a retreat from the effortful goal-oriented state to the biophysically and affectively unstressed default state. Support for this interpretation has been observed in non-fatigued contexts with, for example, less gamma power emerging during rest periods in comparison to active engagement in a cognitively demanding task, i.e., serial math computations (Shivabalan et al., 2020). Flow states provide additional insights into states with low gamma activity. In contrast to the challenging but monotonous tasks producing the vigilance decrement, challenging but engaging tasks lead to a state of “flow” where intensified effort does not elicit an adverse response and withdrawal to the default state (Inzlicht et al., 2018). In a recent video game study examining flow, enhanced gamma power was maintained during high intensity events whereas low intensity events exhibited decreased gamma power (McMahan et al., 2015). In sum, the results suggest gamma oscillations index two functions of the motivational system—effortful and low-effort unstressed states, respectively.

By addressing dissociations between distinct accounts of fatigue, our hybrid framework and computational models add clarity to underlying processes introduced in preceding theories and models. In particular, scaling parameterized motivation by fatigue moderators offer a formal explanation of dynamic state transitions and performance declines across time. Moreover, the underlying processes align with biological-grounded mechanisms, i.e., microlapses approximate disruptions to circadian rhythm dynamics (Gunzelmann et al., 2009a,b) and utility thresholds correspond to activation thresholds for populations of cortical neurons (Helfrich and Knight, 2016). Similarly, parameterizing gamma dynamics in our models increases the fidelity of latent factors driving the compensatory process. As shown in *Model 2*, gamma power continuously reaches unity (increases above baseline) to maintain task performance over time, albeit with reduced efficacy. This pattern is consistent with motivationally-driven compensatory control described by the CCM (Hockey, 1997, 2011).

Our hybrid framework challenges stochasticity assumptions of earlier computational models of fatigue (Gunzelmann et al., 2009a,b; Veksler and Gunzelmann, 2018). In these models, a noise parameter implements the stochasticity observed in behavioral data. By contrast, parameterizing motivation using gamma oscillations resulted in a dynamic response to fatigue without relying on an arbitrary noise mechanism. As shown in Figure 6, this is a key distinction between *Model 2* which uses single-trial gamma power and the CMF which uses fatigue moderators exhibiting a steady monotonic decline over time. In ACT-R, noise is considered a universal feature of cognition rather than specific to a given psychological function (Anderson et al., 2004). However, *Model 2* demonstrates that fluctuations in performance over time can be attributed to a specific set of cognitive processes—in this case, dual motivations interacting with time and microlapses—rather than strictly a function of more generalized uncertainty (i.e., noise) in the decision process.

Though distinct, our hybrid framework overlaps with traditional resource theories which conceptualize performance declines as a function of diminishing resources (Caggiano and Parasuraman, 2004; Warm et al., 2008). Analogously, the hybrid framework views controlled effort as increasingly unavailable when the motivational system favors a low effort, non-aversive state. Indeed, the parameterization of gamma power and the constant utility threshold in our gamma models point to effort as a finite resource used to maintain performance over time—a conclusion consistent with a role for gamma oscillations, as nested in low frequency theta oscillations, in the dynamic allocation of limited resources (Helfrich and Knight, 2016).

Beyond theoretical implications, our hybrid framework offers insights for developing fatigue intervention technologies. One potential intervention involves the use of transcranial direct current stimulation (tDCS) to stimulate neuroplasticity mechanisms by resetting ongoing oscillatory activity. Recently, effective tDCS treatments aligned with changes in gamma activity. For example, gamma power increased in the tDCS treatment group in comparison to a sham treatment group during a vigilance task (Hemmerich et al., 2023). In a clinical setting, tDCS treatment enhanced gamma power coincidentally with improved general cognition and neural connectivity (Liu et al., 2022). Similar tDCS technologies could leverage individual profiles for gamma activity and motivational control to assist professionals performing uninteresting but difficult and high-consequence tasks. Alternatively, gamma power can be used to measure the efficacy of an intervention. For example, high frequency gamma power was assayed to evaluate the improvement of elderly participants' performance in different cognitive training regimens (Akimoto et al., 2016). In a fatigue context, gamma activity could be adapted to index the effectiveness of a motivation intervention over time.

### 7.1 Limitations and future work

The present study had some limitations. Although utility values in our computational models index shifts in the trade-space between motivated on-task and disengaged restful off-task states over time, adding experimental manipulations or including thought probes may have yielded converging evidence for the hybrid framework. For instance, goal setting, rest, feedback, try-harder instructions, and other incentives decreased RTs in comparison to control (no manipulation) conditions (Botvinick et al., 2015; Steinborn et al., 2017; Robison et al., 2021; Schumann et al., 2022; Strayer et al., 2023). Moreover, thought probes frequently (but not always) correlate with motivation manipulations (Herlambang et al., 2021; Robison et al., 2021; Schumann et al., 2022). Fatigue-oriented studies would benefit from routinely incorporating these measures. Other indexes of the motivation trade-space use cortical data acquired by functional magnetic resonance imaging (fMRI) to map state transitions to frontal cortex regions responsible for computing rewards and costs (Langner and Eickhoff, 2013; Kok, 2022). In EEG data, the ERP error-related-negativity (ERN) has been similarly associated with reward predictions and response switching (Yasuda et al., 2004) and affective reward processing mediated by the ACC (Inzlicht et al., 2015). Future studies and computational models of fatigue should consider incorporating an explicit cost-benefit function parameterizing the ERN akin to the utility parameter in our gamma models. Such endeavor would be expected to yield benefits beyond the inclusion of a cost-benefit mechanism. First, the ERN could be construed as representing, at least in part, the biologically-grounded “effort monitor” mechanism described by the CCM (Hockey, 1997, 2011). Second, ERPs record neural activity at a finer temporal resolution (tens of ms) than frequency data (hundreds of ms) in the present study, extracted from a full trial or aggregated across many trials. The increased resolution would allow for identifying the precise timing and neural activity magnitude of state transitions due to the competing motivational drives. Separately, future studies should consider nonlinear approaches to evaluating the theoretical implications of foreperiod and time-on-task interactions given the inadequacies of linear analyses uncovered in recent work (Houshmand Chatroudi et al., 2024).

Though gamma power reliably distinguishes good from poor performers (Herrmann et al., 2010; Kim et al., 2017), our computational models did not evaluate individual performance differences, though previous power (Borghetti et al., 2021) and connectivity (Borghetti et al., 2022) studies have. Our future work includes extending our Gamma models to accommodate these variations in individual subjects' performance.

### 7.2 Conclusion

Our novel hybrid framework of fatigue resolves dissociations between psychological theory, computational mechanisms, and neural behavior, to establish a comprehensive account of time-on-task effects, i.e., the vigilance decrement. Our framework casts motivation as a dual-purpose system balancing effortful goal-oriented control with dynamic transitions to a biophysically and affectively comfortable off-task state. Fatigue moderators, such as microlapses, additionally account for increasing time off-task responsible for performance declines. We parameterized motivation using high gamma power in two computational models to demonstrate the dynamic properties of on-task compensatory control and the off-task default state. We are optimistic that our hybrid framework bridges gaps in the fatigue, motivation, and neuroscience research and contributes to the development of mitigation technologies in affected professions.

## Data availability statement

The datasets presented in this article are not readily available because the current data set has not been approved by the Air Force Public Affairs office for public release. In order to release data, an approval must be obtained. Requests to access the datasets should be directed to lorraine.borghetti.2@us.af.mil.

## Ethics statement

The studies involving humans were approved by Air Force Research Laboratory IRB and University of Dayton IRB. The studies were conducted in accordance with the local legislation and institutional requirements. The participants provided their written informed consent to participate in this study.

## Author contributions

LB: Conceptualization, Formal analysis, Investigation, Methodology, Supervision, Writing—original draft, Writing—review & editing. TC: Conceptualization, Formal analysis, Investigation, Methodology, Writing—original draft, Writing—review & editing. LR: Formal analysis, Investigation, Methodology, Writing—original draft, Writing—review & editing. MM: Investigation, Methodology, Writing—review & editing, Project administration. BV: Writing—review & editing.
